# Hemodynamics effects of recruitment maneuver

**DOI:** 10.1186/cc10715

**Published:** 2012-03-20

**Authors:** D Protsenko, R Magomedov, O Ignatenko, AI Yaroshetskiy, B Gelfand

**Affiliations:** 1 Russian National Research Medical University, Moscow, Russia

## Introduction

Acute respiratory distress syndrome (ARDS) is a frequent complication in critically ill patients. Recruitment maneuver (RM) is a rescue procedure which improves oxygenation [1-3]. However, it is not clear whether improving oxygen delivery (DO_2_) exists after RM. The aim of this study was to evaluate the effects of RM on hemodynamics and DO_2_.

## Methods

A prospective, randomized trial in ARDS patients (AECC criteria). The protocol was approved by the local ethics committee. Fifty-seven patients with extrapulmonary ARDS were randomized into three groups: group A (*n *= 17) - 40 × 40 RM (CPAP 40 cmH_2_O for 40 seconds), group B (*n *= 17) - PCV RM (PIP 40 to 50 cmH_2_O, PEEP 18 to 20 cmH_2_O for 120 seconds), and group C (*n *= 17) - stepwise PCV RM. Gas exchange and systemic hemodynamics by aortal blood flow (transesophageal Doppler; ARROW, USA) were measured before, after, 30 and 120 minutes after RM.

## Results

In all groups we observed rapid increasing of paO_2 _(mmHg) from 65.9 ± 24.9; 77.2 ± 14.0; 87.0 ± 16.7 to 110.3 ± 38.7; 124.5 ± 45.5; 115.2 ± 32.6 (*P *< 0.0001) after RM. We also observed significant improvement of oxygenation 120 minutes after RM (95.6 ± 25.6; 99.3 ± 25.3; 108.1 ± 26.8). There was no statistical difference between groups. Contrarily, DO_2 _(ml/minute/m^2^) after RM statistically significantly decreased from 709.5 ± 297.5; 804.9 ± 217.3; 811.7 ± 638.3 to 569.8 ± 211.9; 675.5 ± 244.7; 661.7 ± 421.3 (*P *= 0.053) and lasted more than 2 hours. The reason for this alteration was decreasing of cardiac output (CO) from 5.3 ± 2.5 l/minute to 3.6 ± 1.7 l/minute (*P *< 0.0001) after RM. We hypothesized that the main reason for decreasing CO is rapid increasing of intrathoracic pressure during RM.

## Conclusion

Three different RMs increase oxygenation and decrease CO equally. But RM does not improve oxygen delivery due to decreasing CO.
